# Mechanisms of Nanoscale Radiation Enhancement by Metal Nanoparticles: Role of Low Energy Electrons

**DOI:** 10.3390/ijms24054697

**Published:** 2023-02-28

**Authors:** Yi Zheng, Léon Sanche

**Affiliations:** Department of Nuclear Medicine and Radiobiology, Cancer Research Center, Faculty of Medicine and Health Sciences, University of Sherbrooke, Sherbrooke, QC J1H 5N4, Canada

**Keywords:** metal nanoparticles, radiosensitization, low-energy electrons, DNA damage, transient anions, dissociative electron attachment, radiotherapy

## Abstract

Metal nanoparticles are considered as highly promising radiosensitizers in cancer radiotherapy. Understanding their radiosensitization mechanisms is critical for future clinical applications. This review is focused on the initial energy deposition by short-range Auger electrons; when high energy radiation is absorbed by gold nanoparticles (GNPs) located near vital biomolecules; such as DNA. Auger electrons and the subsequent production of secondary low energy electrons (LEEs) are responsible for most the ensuing chemical damage near such molecules. We highlight recent progress on DNA damage induced by the LEEs produced abundantly within about 100 nanometers from irradiated GNPs; and by those emitted by high energy electrons and X-rays incident on metal surfaces under differing atmospheric environments. LEEs strongly react within cells; mainly via bound breaking processes due to transient anion formation and dissociative electron attachment. The enhancement of damages induced in plasmid DNA by LEEs; with or without the binding of chemotherapeutic drugs; are explained by the fundamental mechanisms of LEE interactions with simple molecules and specific sites on nucleotides. We address the major challenge of metal nanoparticle and GNP radiosensitization; i.e., to deliver the maximum local dose of radiation to the most sensitive target of cancer cells (i.e., DNA). To achieve this goal the emitted electrons from the absorbed high energy radiation must be short range, and produce a large local density of LEEs, and the initial radiation must have the highest possible absorption coefficient compared to that of soft tissue (e.g., 20–80 keV X-rays).

## 1. Introduction

In the emerging field of nanoscale cancer treatment, metal nanoparticles (MNPs) are expected to play an increasing role in radiation therapy as local radiation dose enhancers, sensitizers, hyperthermia inducers and drug delivery vehicles [1,2]. They could also serve as photosensitizers in treatments with both ionizing UV and non-ionizing radiation [3,4]. MNP therapy offers an alternative strategy to achieve a more localized radiotherapy either by the direct injection of the nanoparticles into cancerous tissue or via selective internalization into the tumor and its metastases [5,6].

The underlying principle of nanoparticle radiation enhancement in cancer cells arises essentially from the production of a huge number of short-range electrons within a microscopic or nanoscale volume. High-Z atoms such as those of MNPs constitute efficient sources of such electrons, which can amplify the induction of lethal lesions in malignant cells and thus the biological efficiency of radiation treatment. The generation of high local density of nanometer range electrons around MNPs is easily achievable with incident 10––80 keV photons, which have mass-energy absorption coefficients larger by about two orders of magnitude than those of biological tissues [7]. The energies of the emitted electrons extend from a few eV up to almost that of the primary photon [8]. However, only electrons emitted from the MNP with energies below about 10 keV have thermalization distances in the nanometer range within biological matter [9].

Many factors can influence cancer treatment with X-ray irradiated MNPs, including irradiation geometry, depth and location of the tumor with respect to the source [10,11,12,13,14,15,16], biodistribution of the nanoparticles inside the tumor volume [17], and the energy distribution of electrons emitted from the nanoparticle. This distribution depends on the atomic number, size, coating and shape of the MNP as well as the energy spectrum of the incident photons [10,11,13]. The photoelectric effect directly prompts the emission of electrons with a wide range of energies that is accompanied by deexcitation cascades in the MNP, where initial molecular orbital vacancies are filled by outer shell electrons, leading to the emission of characteristic electromagnetic radiation and Auger electrons [1,18]. The latter are usually numerous and limited to a range of about 100 nm, over which they can deliver high nanoscopic doses [19]. Auger electrons can scatter within the metal, lose energy and produce via ionization a further generation of lower-energy secondary electrons (SEs). Other short-range electrons can be produced by the absorption of fluorescent photons within the MNP [19,20]. Since some photons and particles emitted from MNPs have ranges larger than a micrometer, not all the energy absorbed from incident X-rays can be retained in a cell and even less within a specific target (e.g., nuclear DNA).

The transport of SEs within the MNP, is subject to various energy-loss processes (e.g., plasmon excitations) [21]. Electrons emitted into the biological medium with energies above the ionization potential can also produce a further generation of low-energy electrons (LEEs) with energies lying essentially below 30 eV [22]. Thus, Auger electrons can destroy cells directly, or via production of numerous SEs both within and around the MNP. The ensemble of these interactions produces a high density of LEEs within a radius of about 100 nm from the center of the MNP, which is almost as intense as that found in ionization tracks of alpha particles [22]. Within their range of only 10 nm, LEEs are highly effective in breaking chemical bonds, producing potentially lethal DNA lesions [23,24,25,26,27].

In this article, we refer to the magnitude of a specific biological or chemical process, resulting from irradiation in the presence of MNPs, to that which occurs without them, as the enhancements factor (EF). Many measurements of EFs reported in the literature confirm the basic principle of nanoparticle radiation enhancement upon X-ray irradiation [21,28]. For example, EFs ranging from 1.27 to 2.56 can be reached with a surprising low cellular concentration of GNPs [29]. Indeed, at concentrations as low as 0.003% of gold per mass of tissue (i.e., percent mass of gold to tissue PMG/T), these nanoparticles produce an EF = 1.25 in 10% survival level of HepG2 cells [29]. For possible translation to human medicine, most simulations use typically 0.1 to 1 PMG/T, which are considered as overrated values [29]. For a homogeneous mixture of water and gold, even coefficients of 0.1–1 PMG/T are not sufficient to produce a significant change of the absorbed dose in biological tissue. It is therefore obvious that it is not the cellular dose enhancement, which produces the increase in tumor regression in MNP-aided radiation therapy. Rather, it is related to the location of the MNPs within malignant cells and the high dose delivered within a nanoscopic volume by extremely short-range electrons.

As shown theoretically by Verkhovtsev et al., high-energy charged particles interacting with GNPs can also generate a considerable number of LEEs [30]. Their yields are about an order of magnitude higher than those from an equivalent water volume and not very dependent on the primary particle energy [30]. Although less efficient than 10–80 keV X-rays by about an order of magnitude, such charged particles produce broadband electromagnetic radiation that can be preferentially absorbed by GNPs. The mechanism of energy absorption is therefore similar to that of photon absorption. Consequently, energy absorption by GNPs from fast charged particles, such as clinical MeV electrons, are expected to arise mostly from the 10–80 keV electromagnetic range. Furthermore, according to Verkhovtsev et al., fast charged particles have considerable probability to excite plasmon modes in GNPs [30], which would also contribute to the EFs ranging from 1.14 to 1.66 observed for cell death and tumor regression after irradiation with high energy electrons and protons [31].

Since DNA damage is expected to have the most detrimental effects on cell survival, the optimal location to irradiate MNPs and obtain high EFs should be within the nucleus, preferably as close as possible to genomic DNA. Some experiments have been performed to increase the transmission of GNPs through the nuclear membrane, by properly conjugating gold nanoparticles with specific peptides, which selectively transports them to the nuclei of cancer cells [32,33]. Considering these advances and the role of DNA as a key biomolecule in radiobiology and the main target in radiotherapy, many studies have focused on the physics and chemistry involved in irradiated DNA in the presence of MNPs or planar metallic surfaces covered with the molecule [1,21,34,35]. They have included experimental and theoretical investigations of the interactions of the Auger and SEs with DNA and its surrounding medium, particularly water, as well as the ensuing production of reactive radicals, including those arising from water and oxygen [36,37,38]. The reactions of such radicals with DNA have often been evaluated, especially within the region around the positively charged MNPs, where the reaction kinetics could be different from those in non-modified cells [36,37,38]. These latter reactions are not considered in the present review.

LEEs can also be produced by UV photons incident on a metal surface or MNP. In this case, the process is simpler, involving only the creation of one positive hole in the metal and a single LEE. Photons of any wavelength up to 600 nm can photo-eject LEEs from metals in water and biological tissue, due to the lowering of the metal work function (WF) relative to vacuum [39]. Photoelectrons of zero to 10 eV can be ejected depending on the metal WF and photon energy. Details are given in Table 1 [40,41,42,43,44,45,46,47]. As explained in the present article, even with energies as low as 0–3 eV, electrons can damage DNA by breaking chemical bonds. Multiple photon excitation with lasers can also lead to photo-ejection of electrons from MNPs [48,49]. This process, which often involves plasmon excitation [30,50], is not covered in this review.

MNPs for the clinic are made of different atoms ranging from Titanium (Z = 22) to Bismuth (Z = 83) [1]. GNPs have probably been the most investigated, due to their useful properties, that include their high atomic number and high density, variable shape and size, as well as the ease of preparation and detection, particularly for imaging, and their ability to bind a variety of ligands [1,21]. These properties also made them ideal candidates for investigating the fundamental mechanisms driving the observed enhancements by MNPs of the chemical and biological effects of high energy radiation. As shown in this article, these mechanisms may become essential in the design and development of MNPs for effective antineoplastic applications.

The present article is focused on the DNA damage produced by LEEs that have been emitted from and surround metal surfaces. In many DNA experiments with LEEs, the target molecules are eukaryotic plasmids [51], which adapt supercoiled configurations similar to the DNA found in human mitochondria [52]. Hence, the studies of plasmid DNA have implications related to radiation-induced damages in both the genome and mitochondria. We take as examples the results obtained with irradiated GNPs bound to DNA, as well as those generated by photoelectrons emanating from gold and tantalum planar metal surfaces covered by DNA. Since LEE emission from irradiated metals is universal, we expect the results described in this article for GNPs, to be at least qualitatively applicable to any other MNP. Details of the energy deposition processes of LEEs, and of their interaction mechanisms are explained with emphasis on the most recent descriptions. Since fairly complete reviews on this topic have appeared prior to 2019, the purpose here is to summarize the most significant progress, and to provide a general and comprehensive overview of the field. Further information on the initial absorption by GNPs of photons of less than 100 keV can be found in the review articles appearing in the book edited by Erno Sajo and Piotr Zygmanski [1]. This book synthesizes the most important advances prior to 2019 in MNP-aided radiation therapy and the microscopic image-based techniques of nanoparticle detection in patients and animals.

## 2. DNA Damage Induced by LEEs in the Vicinity of Irradiated GNPs

LEEs found in biological material surrounding irradiated MNPs arise principally from two sources: emission from the nanoparticle, and from scattering near the nanoparticle of short-range Auger electrons [18,53]. Within biological media, Auger electrons produce ionizations and excitations along their path, which lead to the production of a large numbers (~40 per keV of deposited energy) of cations and SEs [54]. The latter can have energies ranging from zero to several hundreds of eV, but most of them are created with energies below 30 eV [22]. Both the cations and SEs can interact with the surrounding biological matter to produce radicals. Further reactions of these radicals lead to products, which are usually known in the field of radiation chemistry, particularly in aqueous media [55,56,57]. 

**Figure 1:**
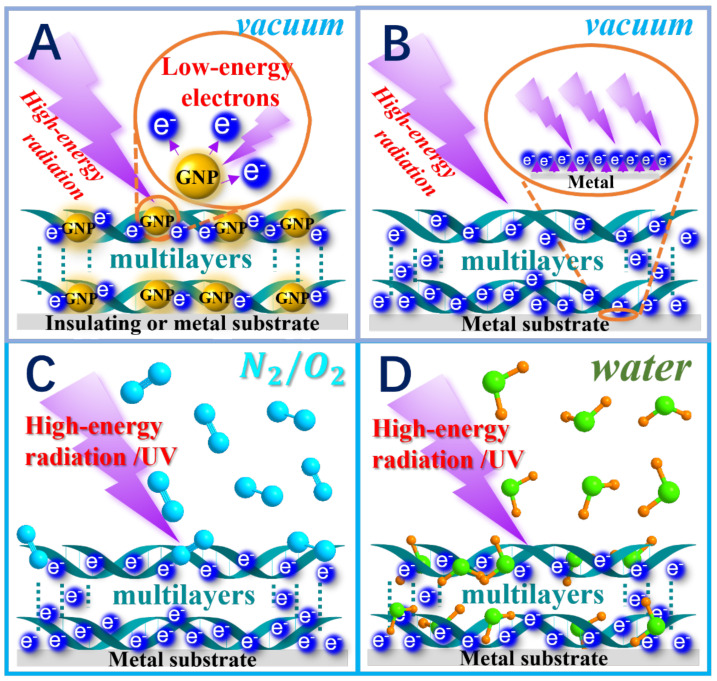
Schematic representation of experiments previously performed with multilayer films of plasmid DNA irradiated with high energy electrons, X-rays or ionizing UV photons. In (**A**), GNPs electrostatically bound to DNA emit Auger electrons that generate short-range (10 nm) LEEs mostly within the multilayer, whereas in other representations, the DNA interact with LEEs ejected from the metal substrate. When the latter is bombarded with 1.5 keV X-rays almost all emitted photoelectrons have energies lower than 30 eV. The multilayer film can be placed in (**B**) vacuum or (**C**) an O_2_ and/or N_2_ atmosphere at standard atmospheric temperature and pressure (SATP). As shown in (**D**), water can be added in experiment C, to produce different humidity levels (Table 2). The added environmental molecules are absorbed within the DNA structure to simulate LEE irradiation conditions of DNA closer to those of the cell.

The ability of irradiated GNPs to amplify the formation of DNA damages has been demonstrated in many investigations [1]. Figure 1A describes schematically a typical experiment, where multilayers of DNA containing GNPs are exposed to high-energy radiation. The EFs vary between 1.3 and 4.8 upon exposure of supercoiled DNA to ionizing radiation in dry films and aqueous solution [34,35,36,58,59]. The yield of a specific damage (i.e., the numbers of damages per electron•molecule) is highly variable, as it depends on the many parameters mentioned in the introduction [34,35,58]. Several studies have compared the yields induced by high-energy protons [60,61,62], electrons [34,63] and X-rays [36,58,59,64,65,66,67,68,69]. Those obtained under vacuum (Figure 1A), from exposure to 60 keV electrons of GNPs (5 ± 2 nm average diameter) electrostatically bound to plasmids in ratios 1:1 and 2:1 in multilayer films produce SSBs and DSBs with EFs of about 2.5 [58]. The yields of these lesions were measured ex-vacuo by agarose gel electrophoresis. In all other experiments with plasmid DNA (i.e., PGEM-3Zf(−)) reported in this article, the yields of conformational damages (i.e., SSBs, DSBs and crosslinks (CLs)) were similarly determined. In all cases, damage yields were measured as a function of either electron, X-ray or LEE fluence, and the exposure-response curves extrapolated to zero fluence. This extrapolation and the low fluences in these experiments ensured that the lesions and their yields did not arise from multiple events [26].

**Figure 2:**
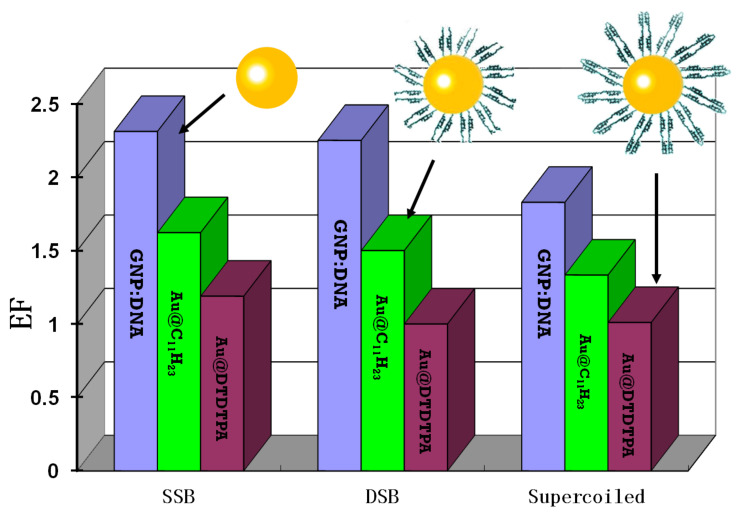
Enhancement factors (EFs) for the formation of single-strand breaks (SSBs), double-strand breaks (DSBs) and loss of supercoiled (LS) induced by 60 keV electrons in GNP–DNA complexes with ratio 1:1 [34]. Each group of three histograms of different colors represent, respectively, SSBs, DSBs and LS. In each group, the EFs were generated by bare GNPs of 5 nm diameter bound to DNA or coated with C_11_H_23_, or DTDTPA (i.e., dithiolated diethylenetriaminepentaacetic) ligands. The corresponding ligand lengths were 2.5 and 4 nm, respectively.

In further experiments with 60 keV electrons, Xiao et al. irradiated 5 nm diameter GNPs coated with ligands of different lengths [34]. These GNPs were mixed in a proportion of 1:1 with plasmids before being deposited on a Ta substrate. The EFs diminished from 2.3 to 1.6 and 1.2, as the length of the ligand varied from 0 to 2.5 to 4 nm as shown schematically in Figure 2 [34,70]. In the experiments of Spaas et al., with 200 keV X-rays irradiating 5–30 nm diameter PEG-coated GNPs, a larger difference in ligand length (∼11 nm) was needed to observe a similar reduction in EF (60%) [68]. In both cases, attenuation by the coating of the number of short-range LEEs reaching DNA was proposed to explain the decreasing radiosensitization with increasing ligand length. From this perspective, the highest levels of radiosensitization by GNPs should be achieved with the shortest possible ligand and a partially covered nanoparticle surface. Presently, many, but not all (e.g., nucleus-penetrating peptide) DNA-binding ligands may be too long for the LEEs emitted from the GNP to reach the helix with sufficient energy to produce lethal damage [32,33,71]. However, a non-negligible portion of the LEEs from the distribution produced by the emitted 100 nm-range Auger electrons during 10–80 keV photon irradiation should be capable of reaching and damaging genomic DNA in the presence of much longer ligands [72,73]. In any case, only partial covering of GNPs by the ligand appears as a favorable condition to increase Auger and LEE mediated DNA damage.

Even though strand break assays are highly sensitive, they give little information on the nature of the reactive species and exact chemical modifications to the structure of DNA. Recently, Huwaidi et al. identified and quantified several individual modifications resulting from exposure of dry films of calf-thymus DNA to 10–80 keV X-rays in air, in the presence of GNPs of 5 nm average diameter [74]. From analysis of the damage by ultrahigh performance liquid chromatography coupled to tandem mass spectrometry, they identified the formation of three major types of degradation products: (1) the release of nonmodified nucleobases, (2) the formation of reduction and oxidation species, and (3) the formation of products arising exclusively from the reaction of LEEs with DNA. The yields of these products as a function of the dose of 10–80 keV X-ray radiation absorbed by the films were measured [74]. The major damage was release of four nucleobases, while eight modifications of 2-deoxyribose moiety were detected including dideoxynucleoside (ddN products consisting of four nucleosides with an intact nucleobase and modified 2-deoxyribose moiety with C3′−O and C5′−O groups transformed to the corresponding dideoxy derivatives (2′,3′- and 2′,5′-ddNs) [74]. These products can only be induced by reactions of LEEs with DNA and hence testify that such electrons were produced from energy absorption by the GNPs [75,76]. Furthermore, ddN yields increased as a function of the ratio of GNPs to DNA and reached a plateau above 1% ratio of the moles of GNPs per mole of nucleotides. The reduction products were identified as 5,6-dihydro-2′- deoxyuridine and 5,6-dihydrothymidine, and the oxidation products as 8-oxo-7,8-dihydro-2′-deoxyguanosine and 5-hydroxymethyl-2′-deoxyuridine. In units of lesions per million DNA bases per Gy, the yield of base release was the highest (61.6) followed by that of reduction (7.7) and oxidation products (8.6) [74]. Although the ddN products served to establish that LEEs were implicated in damaging DNA, their yields were much lower (0.53) than those of the other products. Both the release of intact bases and the formation of ddN products could be related to initial temporary electron capture at different position within the DNA helix. [77,78] The ensemble of the results of Huwaidi et al., their measurements as a function of dose and GNPs concentration, as well as comparisons with direct electron impact data reported in Section 5.2, provided considerable evidence on the role of LEEs [74]. These authors strongly suggested that most of the measured degradation products arose from reaction with DNA of the copious number of LEEs generated by the short-range Auger electrons emitted by the GNPs upon X-ray irradiation. Finally, we note that even without the production of SEs, electrostatic binding of GNPs to plasmids, enhances their sensitivity to LEE impact [35].

## 3. DNA Damage Induced by LEEs Emitted from a Metal Surface in Various Environments

Most of the experiments described in this section correspond to those described schematically in Figure 1B–D. When plasmid DNA was deposited on a clean gold substrate and bombarded with 60 keV electrons (Figure 1B), the damage yields were an order of magnitude higher for 10 nm coverage, compared to that measured with 2.9 μm thick films [58]. In a thick film, most of the photon energy is absorbed by the film, whereas in a 10 nm thin film, much of the photon energy is absorbed by the gold substrate. When exposed to 60 keV electrons, the gold surface emits mostly SEs of energies lying below 30 eV [79]. Thus, the order of magnitude enhancement in damage yields, when going from thick to thin films, was related to the higher effective density of LEEs in the 10 nm film due to SE emission from gold [58]. This result testified to the higher bond-breaking efficiency of LEEs generated close to metallic surfaces relative to that of the incident photons.

Further experiments were performed with multilayer films of plasmid DNA deposited on metal substrates irradiated by X-rays, either in vacuum or under standard atmospheric temperature and pressure (SATP). In the initial vacuum experiments of Cai et al., a tantalum (Ta) surface irradiated with 1.5 keV AlKα X-rays, was alternatively covered with monolayer (ML) and thick (20 µm) DNA films [80]. These two different thicknesses allowed the damage induced by photoemitted electrons to be compared to that produced by the X-rays. The emitted LEEs had an average energy of 5.8 eV and a distribution peaking at 1.4 eV [80]. The G-values (i.e., yields of a given damage per energy deposited) for SSBs and DSBs induced by LEEs were 86 ± 2 and 8 ± 2 nmol/J, respectively [80].

Following these investigations, an improved method (Figure 1C,D), suitable for irradiation of thin biomolecular films deposited on a clean metal substrate surrounded by a pure gas, vapor, or mixture thereof, at SATP was developed to better simulate cellular conditions [81,82,83,84]. It was used to measure damage induced to plasmids by photoelectron emission from a Ta surface irradiated with 1.5 keV AlKα X-rays [81,82,83,85,86]. The plasmid films were irradiated under otherwise identical experimental conditions in air [80,85], nitrogen/oxygen [81], mixtures of N_2_ and O_2_ (Figure 1C) [83], with humidity levels varying from 0% to 100% [82,83] as depicted in the drawing of Figure 1D. The G-values for LEEs were determined by subtracting the damage yields measured with the plasmids deposited on a glass substrate from the yields obtained with the same amount of DNA covering the metal surface. The yields varied depending on the perturbation of the DNA by the environment and the reactions with DNA of the radicals produced by LEE interactions with the surrounding molecules [86,87]. These radicals were expected to be much more numerous when an appreciable number of environmental molecules were absorbed by the DNA (e.g., the production of OH radicals from water absorbed by DNA at high humidity levels) [82]. The studies clearly illustrated that the hydration level and the oxygen content of the surrounding atmosphere increased yields of LEE-induced DNA damage, and consequently, the corresponding G-values, as seen from Table 2. The G-values listed correspond to those for SSBs (G_SSB_), DSBs (G_DSB_) and loss of supercoil configuration (i.e., total conformational damage, G_LS_) induced by 1.5 keV X-ray photoelectrons emanating from a gold or tantalum substrate covered with 5 MLs of plasmid DNA [80,81,82,83,84,85,86,87].

**Table 2:** Comparison of G-values for SSBs (G_SSB_), DSBs (G_DSB_) and loss of supercoil (total damage, G_LS_) induced by 1.486 KeV X-ray photoelectrons emanating from a metal substrate covered with 5 MLs of plasmid DNA.

	Substrate	Average LEE Energy (eV)	G_LEE_ (×nmol/J)			Hydration Level ^a^	Environment
			**G_SSB_**	**G_LS_**	**G_DSB_**		
Brun et al. [85]	Gold	4.0		400 ± 200		*Γ* = 2.5	vacuum
	Gold	4.0		600 ± 200		*Γ* ≈ 10	Air
Alizadeh et al. [82,86]	Ta	5.85	248 ± 65	260 ± 50		*Γ* = 2.5	N_2_, SATP ^b^
	Ta	5.85	226 ± 59	247 ± 64	-	*Γ* = 5 ± 1	N_2_, SATP
	Ta	5.85	223 ± 57	309 ± 80	-	*Γ* = 10 ± 1	N_2_, SATP
	Ta	5.85	268 ± 70	412 ± 107	21 ± 5	*Γ* = 20 ± 1	N_2_, SATP
	Ta	5.85	271± 140 ^c^	420 ± 277 ^c^	21 ± 5	*Γ* = 33 ± 1	N_2_, SATP
Alizadeh et al. [81]	Ta	5.85		227 ± 15		*Γ* = 2.5	N_2_, SATP
				415 ± 15		*Γ* = 2.5	O_2_, SATP
Alizadeh et al. [81,83]	Ta	5.85	206 ± 54	288 ± 75	10 ± 3	*Γ* = 2.5	N_2_ + O_2_, SATP
	Ta	5.85	432 ± 112	473 ± 123	-	*Γ* = 2.5	O_2_, SATP
Alizadeh et al. [82]	Ta	5.85	540 ± 80	737 ± 110	46 ± 66	*Γ* = 2.5	N_2_O, SATP

^a^: *Γ*, moles of water per mole of nucleotides. ^b^: standard atmospheric temperature and pressure. ^c^: corrected results.

**Figure 3:**
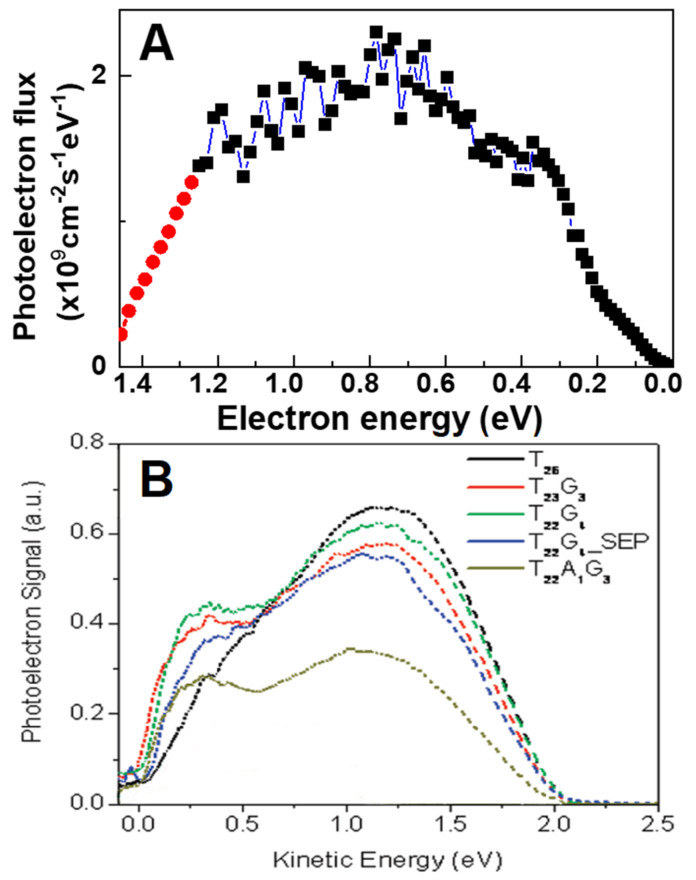
(**A**) The energy distribution of photoelectron flux calculated with Folwer’s law with the work function of 3.7 eV for the Ta−DNA surface irradiated with Xenon lamp [41] Copyright 2016 by American Chemical Society. (**B**) Energy spectra of photoelectrons transmitted through self-assembled monolayers of oligonucleotides of various compositions and sequences anchored to a gold surface. The photoelectrons were emitted by 6.42 eV photons striking the surface. The oligomers are anchored to the substrate by a thiolate terminal group [89]. Each transmission spectrum from different DNA oligomer is represented by a different color. T_22_G_4_-SEP indicates that each of the four guanines (G) is separated by 3 thymines (T), while in the other oligomers the Gs are clustered. Adapted from [89].

To our knowledge, only Liu et al. measured the DNA damage induced by photoelectrons emanating from a metal surface at SATP that were created by single UV photons [41]. SSBs were induced in 5 mL thick plasmid DNA films deposited onto a polycrystalline Ta surface or that of a cleaned commercial foil. Photons in the range of 240–400 nm were produced by a filtered Xe lamp and the samples were irradiated in dry nitrogen at SATP. For each bare and sample-covered substrate, the WFs were measured with a Kelvin probe, and the voltages were referred to the WF of polycrystalline Ta of 4.12 ± 0.05 eV in vacuum. The energy distribution of emitted electrons lied between 0 and 1.5 eV as calculated using Fowler’s law: Y = α (hν-WF)^2^, where Y is the quantum photoelectron yield, hν the photon energy, and α a material specific constant (e.g., 4.2 × 10^−4^ for polycrystalline Ta) [88]. The distribution of photo-ejected LEEs from a polycrystalline-Ta-DNA surface in gaseous molecular nitrogen is shown in Figure 3A. After irradiation, only SSBs were detected by electrophoresis, indicating that 0–1.5 eV electrons cannot induce DSBs. The yield of SSBs due to LEE interactions in the films was obtained by subtracting from yield recorded with the metal substrate, the value recorded on glass; the latter does not emit a significant number of photoelectrons. The G values for SSBs and total conformational damage induced by LEEs to plasmids were 47 ± 37 and 49 ± 38 nmol J^−1^, respectively [41].

The group of Naaman measured the low energy photoelectron current emitted in vacuum by 6.42 eV photons incident on a gold substrate, covered by self-assembled MLs of short DNA oligomers [89,90]. These MLs were anchored to the substrate by a terminal SH group. The transmitted photocurrent was measured, and analyzed in energy, taking into consideration the modification of the substrate WF by the oligonucleotides. For a given film, the transmitted electron current at any energy was related to the inverse of the electron capture probability of molecules forming the film. The results obtained with self-assembled MLs of different bases and sequences are shown in Figure 3B; each film characteristic is given in the upper right corner [89,90]. The oligonucleotides were mainly composed of thymine bases, with guanine (G) and adenine inserted at certain positions. The Gs were assembled into clusters of 3–4, except for one SAM designated as SEP in Figure 3B, where four Gs were separated by 3 thymine bases. These G substitutions decreased the transmitted LEE current as seen from Figure 3B. Moreover, the energy distribution exhibited a characteristic dip around 0.6 eV in the photoelectron transmission probability of the oligonucleotide films. This reduction in transmitted current can be interpreted to reflect an increase of the electron capture probability at this energy, which is dependent on the number of G bases and their clustering level. Interestingly, addition of a single adenine base adjacent to a 3G cluster, dramatically reduced the LEE current (i.e., it increased electron capture). In other experiments, transmission spectra were recorded with SAMs of double-stranded DNA of the same length as the singly stranded oligomers [91,92]. The former captured electrons less efficiently than the single strands. All experiments provided evidence that electrons emitted from a gold surface can be captured by the nucleotides or their fundamental units with different probabilities, depending on base sequence [89,90,91,92]. The results also indicated that Gs are most effective in stabilizing captured electrons [89,90,91]. They were interpreted via the mechanisms explained in Section 5.

## 4. Enhancing Radiation Damage and Its Biological Effects by Combining Platinum Chemotherapeutic Agents (Pt-CAs) and GNPs

Enhancements in radiation damage and biological effect due to the respective chemical and electrostatic binding of Pt-CAs or GNPs to DNA, have been have demonstrated in several studies, both in vitro and in vivo [93,94,95,96,97,98,99,100,101,102,103,104]. After comparing the independent effects of these agents under 60 keV radiation, it became obvious that combining them within a common carrier could considerably enhance the benefit of chemoradiation therapy and/or MNP-aided radiation therapy, particularly if the combined agents could enter the nucleus of cancer cells and lie close to DNA. In such a situation, the GNPs would produce large quantities of LEEs that would strongly react with DNA already sensitized by the Pt-CAs. With Pt-CAs or GNPs bound separately to DNA, 60 keV electron damage increased by factors of 2–4 [34,58,63,93]. However, when subjected to the same experimental conditions, DNA irradiation in the presence of both GNPs and Pt-CAs increased DSBs by a factor of 7.5 [93]. This last observation led to a series of in vitro and in vivo experiments to evaluate the antineoplastic potential of this combination [105].

Charest et al. assessed the synergy in cancer cells and malignant tumors, between 80-keV X-ray irradiation and GNPs administered together with Pt-CAs [105]. To increase the probability of the local combination of GNPs and Pt-CAs within tumor cells, the two compounds were encapsulated in a liposome capable of fusion with cellular membranes. The HCT116 human colorectal cancer cell line was chosen for in vitro clonogenic experiments with carboplatin and oxaliplatin as the CA. The tumor response was determined in immunodeficient NU/NU nude mice implanted with a HCT116 tumor and the CA carboplatin [105]. The liposomes were administered by convection enhanced delivery [106]. In both types of investigations, the results obtained with radiation and the liposomal combination of Pt-CA and GNPs were compared to those generated under the same conditions with no encapsulation. Moreover, control experiments were conducted with radiation alone and irradiation of each individual component of the liposomes and their binary combination. In all experiments, the dose of each of components was varied.

The most promising results of the clonogenic assays were obtained with a low dose of encapsulated GNPs and carboplatin irradiated with 2 Gy of 80 keV X-rays [105]. In this case, the surviving fractions were much lower compared to other combinations of the equivalent amounts of the compounds in non-encapsulated delivery. As expected, the most effective radiotherapeutic treatment of NU/NU mice was obtained when carboplatin and GNPs were simultaneously incorporated into tumor cells via the liposomal carrier. Although much more information is needed to explain the metabolic and cellular behavior of this liposomal cancer treatment, the encouraging outcomes stressed the potential benefits of MNP-aided concomitant chemoradiation therapy and the development of new antineoplastic drugs composed of MNPs co-encapsulated in liposomes with Pt-Cas [105]. The studies for synergy of GNPs with other antineoplastic drugs can be found in recent reviews and publications [73,107,108,109].

## 5. Mechanisms of Action of LEEs

As seen from the results presented in Section 2 and Section 3, LEEs ejected from irradiated GNPs and metal surfaces can induce SSBs, DSBs, CLs, electron stabilization and the formation of reduction, oxidation and ddN products in DNA. What is most surprising is the ability of 0–1.5 eV LEEs photoemitted from a Ta surface to produce SSBs [41], considering that it takes about 5 eV to break a bond within DNA. The mechanisms responsible for all these observations are described in this section.

The dynamics of LEE scattering within biological material must be described in terms of wave functions [110,111]. In large biomolecules, LEE wavelengths are commensurate with the distances between the constituent building blocks of DNA, such as the nucleotides [112]. Hence, intra- and inter-molecular coherent scattering modulate electron energy losses and bond dissociations [84,113,114,115]. Even in cells, where the apparently random orientation of biomolecules could destroy long-range coherence, constructive interference of electron waves can persist, as shown in amorphous ice [116]. In the case of the DNA molecule, LEEs are expected to first diffract within the chains of quasi-evenly spaced nucleotides [117]. Afterwards, they usually localize on the bases forming transient anions (TAs) [112,113,118].

It is well-known that LEEs interact with isolated or condensed molecules either directly or via the formation of Tas [110,114,118,119]. The direct interaction occurs at all energies because the potential between the electron and the molecule is always present. The electron energy dependence of any direct process exhibits a smooth, featureless increasing signal as a function of electron energy. In contrast, when a molecule temporarily captures an incoming LEE into an additional orbital, the resulting TA can enhance inelastic scattering and/or product yields at the energy of this orbital [110,112,114,120]. In other words, the yield function of a specific product or energy loss process that is modulated by a TA, usually exhibits a pronounced maximum at the TA energy, superimposed on the smaller background signal arising from the direct interaction.

Electron resonances (i.e., the formation of TAs) and their decay into various channels (i.e., elastic scattering, molecular excitation and dissociation) have been amply described and reviewed in the literature [84,110,114,120,121,122,123,124]. The perturbating effects of water on such resonances and their decay channels has also been reviewed, with emphasis on theoretical progress [125]. A TA has an intrinsic width in energy that depends on its lifetime as dictated by the uncertainty principle. This width can be estimated from the shape of the maximum in the yield function. Essentially all molecules, from hydrogen up to those as large as genomic DNA can form TAs by interacting with LEEs. In DNA, TAs result from temporary electron capture by the phosphate group or a base, within any of the nucleotides and control damage yields below 15 eV [112,113,114,115,116].

There are two major types of Tas: shape and core-excited resonances. The former occurs when a LEE temporarily occupies an otherwise unfilled orbital of a molecule in its ground state [110,112]. Core-excited resonances or ‘two-particle, one-hole’ states result from electron capture by the positive electron affinity of an electronically excited state of a molecule, or subunit of a large biomolecule (e.g., a DNA base). Both shape and core-excited resonances can autoionize (i.e., re-emit the electron) leaving the target molecule (or site) in the ground state or an excited rotational, intramolecular, or intermolecular (i.e., phonon) vibrational mode. In addition, core-excited resonances can decay into electronically excited states.

Under certain conditions, a TA can dissociate into a neutral radical and a stable anion. This occurs when an anion state has one or many repulsive orbitals and its lifetime is of the order of, or larger than that the time of bond rupture along one or more of its dissociative coordinates. The process is called dissociative electron attachment (i.e., DEA) [126]. If the lifetime is too short the additional electron leaves before dissociation of the TA. Competition between dissociation and autoionization of a TA depend exponentially on its lifetime [110,119,127]. This relationship makes the magnitude of the decay channels of TAs very sensitive to the environment [114]. We therefore expect surrounding atmospheric gases, liquids or solids (e.g., MNPs) to modify these decay channels. This phenomenon has been observed in various experiments with relatively small molecules [110,120] and in DNA, as reported in Section 3.

### 5.1. Shape Resonances in DNA

Within DNA, shape resonances are formed by temporary electron capture into a previously unfilled orbital of a base or phosphate group [112,128]. A priori, shape resonances have four major decay channels: (1) re-emission of the captured electron without energy loss, (2) re-emission of the electron with vibrational and phonon excitation energy losses, (3) DEA, and (4) resonance stabilization. The results obtained with self-assembled MLs of different bases shown in Figure 3B [89,90], can be explained by process (4). Here, the TA formed on one of the bases, exists for a time much longer that the vibrational periods of the nucleoside constituents. Under such a condition, vibrational energy transfer between these constituents becomes possible. At the limit of high vibrational loses by the TA, the extra electron reaches the anion ground state and is trapped by one of the bases having a positive electron affinity. As seen in Figure 3B, this process is highly dependent on sequence and number of G bases in a DNA strand. In decay channels (1) and (2), the captured electron is likely to be re-emitted into the strand, scattering and diffracting along the helix [113].

Below 5 eV, repulsive TA states were found to be sufficiently long-lived to dissociate via DEA [110,128]. Most of them are formed by electron capture into a previously unfilled π* orbital of a base [129], but this orbital can mix with σ* states of the same base, as determined theoretically [13]. The latter authors provided the potential energy surfaces in the ground and vertical excited states for the C1′−N bond within the ribothymidine anion in a dry vacuum and in solution [129,130,131]. A doublet π-σ* state could mix with a dissociative doublet σ* state around 1.3 eV, to permit an essentially barrierless C1′−N bond dissociation and hence nonmodified nucleobases release. This energy is close to that of 1.2 eV observed experimentally by Ptasinska et al. for gas-phase thymidine breakage of the C1′−N bond [132]. These results can be compared to those recorded from thin films of 16-mer oligonucleotides (comprised of an equal number of the 4 different DNA bases) that were irradiated with a beam of 1.3 to 2.3 eV electrons [133]. At these energies, damage can only be induced via the decay of shape resonances into various DEA channels. The distribution of the products from these irradiations, detected by liquid chromatograpy/mass analysis, where similar to those observed with the presence of GNPs [74]. By far, the most abundant yields (75–85%) arose from unaltered base release via C1′−N bond scission, as predicted theoretically [130]. The other degradation products were reduction (14–23%) and ddN (1.4–2.1%). These results obtained with uniquely LEE impact, sustain the previous interpretation of results from 10–80 keV X-ray irradiation of GNPs with DNA, i.e., that most of the damage to DNA covering GNPs arise from LEE interactions [74].

**Figure 4:**
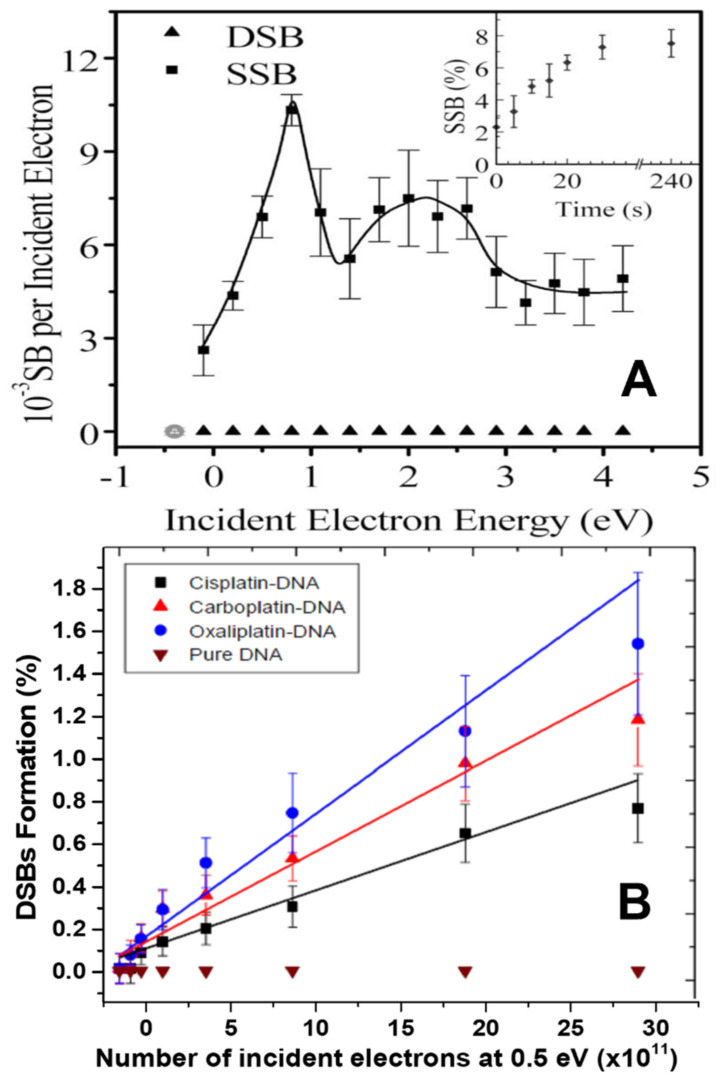
(**A**) Yields of DNA single strand breaks (SSBs) and double-strand breaks (DSBs) vs. incident electron energy. The inset shows the dependence of the percentage of SSBs on irradiation time for 0.6 eV electrons [128]. Copyright by American Physical Society. (**B**) Fluence-response curves for LEE-induced DSBs in 5-ML Pt-CA-plasmids and unmodified plasmid films induced by 0.5 eV incident electrons, which are adapted from [139]. The ratio of Pt-CAs to DNA was 2:1.

Beside DEA on a base, a captured electron can transfer from a base to an adjacent phosphate unit in DNA before cleavage of any bonds [134]. In this case, DEA can occur on the phosphate group, breaking the C-O bond at the 3′ or 5′ position. Such electron transfer can be seen as a crossing between the extra-electron π* orbital of the base and a low-lying σ* orbital of the phosphate group [135,136,137]. First predicted theoretically by Barrios et al. [138], this phenomenon was observed experimentally two years later by Martin et al. [128]. The SSBs yields produced by single electrons, impinging on condensed films of plasmid DNA, deposited on a Ta substrate in ultrahigh vacuum (UHV) are shown in Figure 4A as a function of electron energy [128]. The energy dependence of the SSB yields exhibits two maxima at 0.8 and 2.2 eV. This yield function could be correlated to that of anion radicals arising from DEA to gaseous nucleotide [78] and thus support the electron transfer mechanism postulated by Barrios et al. [138]. Interestingly, the maximum in Figure 4 at 0.8 eV lies close to the dip at 0.6 eV in Figure 3B, indicating that the same shape resonance may have two electron transfer pathways: one to the phosphate group, and another one to another site along the oligonucleotide, where the electron stabilizes. Here again, a similarity exists between the results obtained by photoelectron emission from a gold surface and those produced directly by electron impact.

As seen from the bottom curve in Figure 4A, 0–5 eV electrons did not induce DSBs in the plasmids. Similar results were reported from measurements of 2 to 20 eV yield functions of non-DSB cluster damages [26]. However, 2 eV electrons have been shown to induce crosslinking between plasmids. We can therefore conjecture that the initial radicals that produced this sort of CLs could also be effective in cells to bind DNA with proteins and hence create potentially lethal lesions [26]. Moreover, in the presence of Pt-CAs 0–2 eV electrons can induce clustered lesions in plasmids (i.e., DSB and non-DSB cluster damage) [139,140]. The most striking results were those obtained by Rezaee et al. with 0.5 eV electrons incident on 5 mL plasmid films [139]. The fluence-response curve of the yields of DSBs recorded with such electrons is presented in Figure 4B. No DSBs are observed in irradiated unmodified DNA, but as obvious from the other curves, 0.5 eV electrons are capable of inducing DSBs, when cisplatin, carboplatin and oxaliplatin are bound to DNA. These results have been explained by the formation of a single shape resonance, where the electron wavefunction splits between two unfilled orbitals of the Pt-CA linking two opposite DNA strands [139]. These experiments clearly established that, with a single interaction, 0–2 eV electrons can induce cluster damages in Pt-CA-DNA complexes. Thus, irradiated MNPs emitting LEEs in this energy range close to DNA bound to a Pt-CA can produce damage potentially lethal to cell survival [105,140]. This explanation provided a fundamental mechanism related to the results described in Section 4.

### 5.2. Core-Excited Resonances in DNA

Near and above the energy threshold (~5 eV) for electronic excitation [141], LEE interaction with DNA can lead to the formation of core-excited resonances (i.e., core-excited TAs), up to energies of about 15 eV [26,84,112,142]. UV or X-ray generated photoelectrons emitted from MNPs or produced nearby usually have energy distributions lying within this range [21]. Thus, core-excited TAs are also expected to play a role in damaging DNA lying close to irradiated metals and in MNP-enhanced radiotherapy.

**Figure 5:**
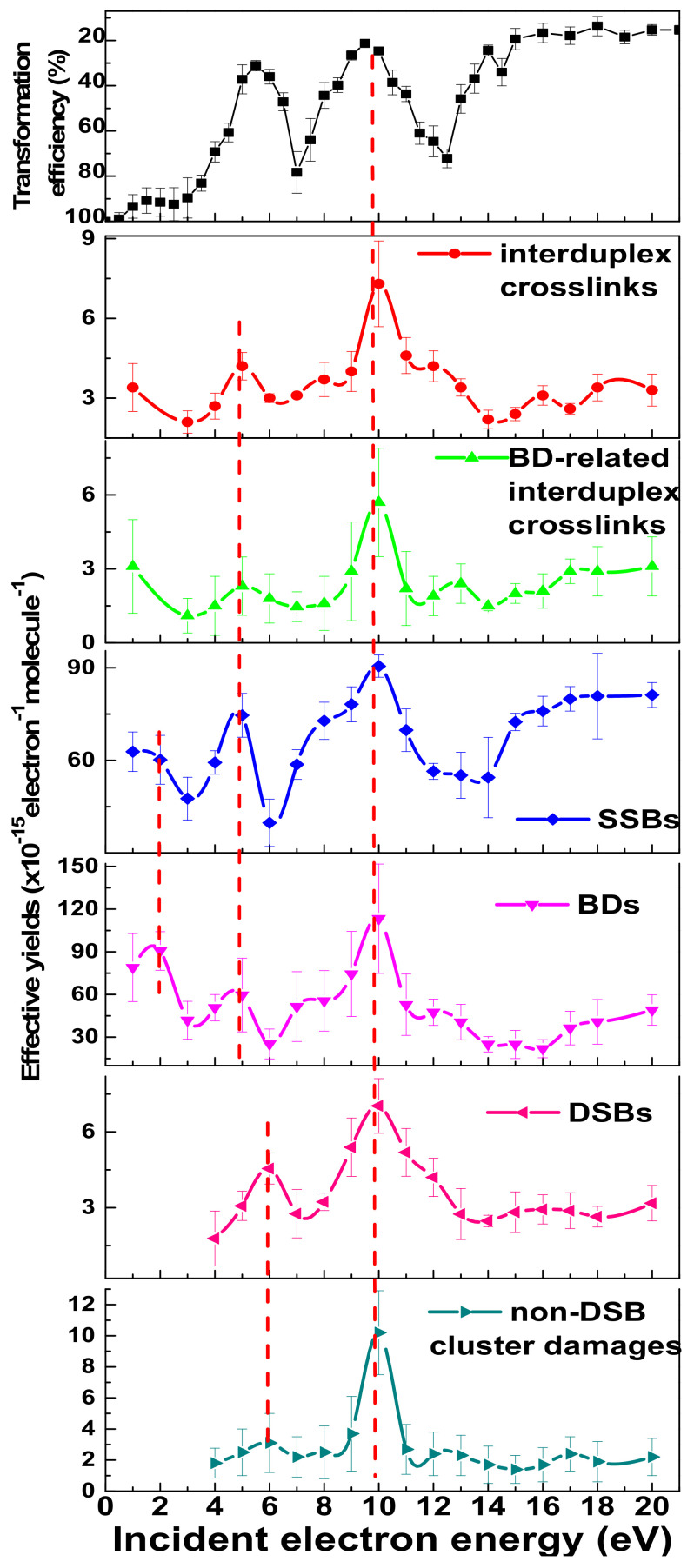
The upper frame is the complement of the transformation efficiency of JM109 *E. Coli* cells, containing PGEM3Zf(-) plasmids bombarded with LEEs of 0.5 to 21 eV [143]. It is compared to the single-electron yields of interduplex and BD-related interduplex crosslinks, SSBs, BDs, DSBs, and non-DSB clustered damage, recorded as a function of electron energy [26]. The dotted lines indicate the energies of TAs at 2, 5, 6, and 10 eV [142].

Like shape resonances, core excited TAs can be detected as maxima in the yield function of various damages. Examples are shown in Figure 5, which exhibits yield functions obtained under single LEE-collision conditions in ultra-high vacuum. The plasmid films of 5 mL thickness were deposited on a Ta substrate [26]. As previously mentioned, conformational damage (SSB, DSB and CLs) was analyzed outside vacuum, by electrophoresis gel [26,51]. Base damage (BD) was revealed and quantitated by treating the irradiated samples with enzymes capable of transforming a BD to a SSB. In this manner, the yield functions of BDs, BD-related CLs and non-DSB cluster damages could be generated [26]. The curve on top of Figure 5 was obtained in a different, more biological experiment, which measured the transformation efficiency of *E. coli* bacteria [143]. The latter were incubated in an antibiotic-rich environment that would otherwise destroy them. However, in this experiment, a plasmid encoding an enzyme capable of inactivating the antibiotic was transferred into the cells, so that they could survive [143,144,145]. When the encoding plasmid was pre-irradiated with LEEs prior to transfer into *E. coli*, the cell resistance to the antibiotic was reduced due to LEE-induced damage, and lower transformation efficiency of the bacteria observed. The complement of *E. coli* survival (i.e., the complement of the transformation efficiency) and its dependence on the energy of LEEs incident on the injected plasmid is shown on the top of Figure 5.

In all the curves of Figure 5, the maxima at 5 and 10 eV are caused by core-excited TAs decaying into the BD, SSB and CL channels [26]. Those located at 6 and 10 eV arise from decay of core-excited TAs in the DSB and non-DSB clustered damage channels [26]. These damages arise from two or more simple lesions, within one or two turns of the DNA helix caused by an initial single electron capture [27,142]. The increase at 2 eV in Figure 5 in the SSB, BD, and CL yield functions is due to the formation of a shape resonance, as explained in Section 5.1 [128]. The peaks in the clustered damage (i.e., DSB and non-DSB) yield functions lie at about the same energy as those in the complement of the *E. coli* survival function shown on top of Figure 5 [143]. This coincidence in energy indicates that clustered damages induced by LEEs can modify the biological function [26]. This is no surprise, since it has been widely demonstrated that unrepaired DNA clustered lesions in cells are responsible for mutagenic, genotoxic, and other potentially lethal effects of ionizing radiation [142,146,147,148,149]. The results reported in this subsection further demonstrate that potentially lethal lesions created by the interaction of a single LEE can arise from the decay of core-excited TAs into multiple bond-breading channels. In other words, the numerous LEEs produced by high energy radiation interacting with MNPs can directly cause cytotoxic lesions without the intervention of the radicals produced by cations and SEs [26].

**Figure 6:**
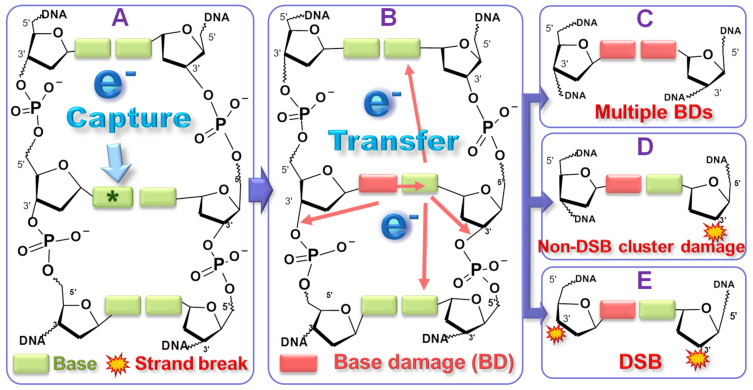
Diagram of the reaction pathways for a single LEE to produce all observed types of DNA damage. Possible electron transfer sites are indicated by red arrows. The electron (e^−^) is initially captured by a base, forming a core-excited transient anion (**A**), where DEA can occur producing a base damage (BD). Alternatively, the electron can transfer to the phosphate group to produce a SSB. The captured electron can also detach from the base anion, leaving the base in a dissociative state (**B**). The base is damaged and the detached electron transfers to other sites where DEA occurs (B). Transfer to the opposite base can result in two adjacent BDs (**C**), whereas transfer to the phosphate unit in the same or opposite strand, can cause a strand break via C-O bond breakage (SSB via DEA + BD) (**D**). A DSB can be produced, if the BD on the left strand is converted to a strand break (**E**). Electron hopping between bases can create a BD or SSB farther away from the initial electron capture site [118]. (Copyright 2021 American Chemical Society).

A general mechanism explaining the behavior of the damage yield functions (Figure 5) in the energy region of core-excited TAs is depicted in Figure 6, using a schematic diagram of a short portion of the DNA double helix [26,150]. Frame A on the left represents an energetic electron being captured by a base via the formation of a core-excited TA. The later can decay in several ways. In the simplest case, the TA dissociate (i.e., DEA) causing BD or non-modified base release; these are the most prominent lesions. If the electron transfers from the base to the phosphate group, a TA can form at that site, and its dissociation can lead to a SSB. In the middle frame (B) an electron from a core-excited TA leaves the base in an electronically excited state, which dissociates causing a BD. The departing electron can transfer to a nearby site (in the same or opposite strand) or to a more distant site, via hoping along either chain. Another TA can form at this second site, where it can decay by DEA. This mechanism, producing two damaged sites with a single electron, can thus explain the observation of clustered damage in a single-electron yield function. Depending on the capture of the transferred electron by a base or a phosphate group, two different types of cluster damage become possible, as shown in frames C and D on the right in Figure 6. These include (C) double BD and (D) a strand break with a BD. If a BD in C or D is converted to a strand break by the formation of dideoxynucleoside products (2′,3′- and 2′,5′-ddNs), then a DSB is formed, as represented in frame E [134,151]. By mechanisms C, D and E, cluster lesions that are potentially lethal to cells can be embedded into DNA molecules lying close to irradiated MNPs.

## 6. Conclusions and Future Challenges

We have shown in this article that the numerous LEEs produced near irradiated MNPs or planar metal surfaces are highly efficient in damaging DNA. These LEEs can be produced by electromagnetic radiation or particles of any energy, greater than that required to emit electrons from the irradiated metal. Emitted low energy and Auger electrons can produce a second generation of LEEs, via their interaction with biological matter beyond the metal. This radiation-induced electron emission results in a high density of LEEs within about 100 nm from the metal surface. Considering their short range (~10 nm), LEEs likely have the greatest potential to enhance local nanoscale radiotherapy and phototherapy, when the MNPs can be delivered to the nucleus of cancer cells, preferably as close as possible to genomic DNA. In fact, the range of LEEs is approximately four times the diameter of the DNA helix and hence they can deposit a large amount of their energy into the molecule, if located in its immediate vicinity. Furthermore, electrostatic binding of MNPs to DNA modifies its chemical structure and/or morphology, inducing radiosensitization independently of electron emission [152].

Considering ongoing refinements in the methods of targeting MNPs to cancer cells via intravenous injection of targeting carriers [57,97,153,154] or convention enhanced delivery (i.e., direct intratumoral injection) [104], these particles could become highly effective in nanoscale radiotherapy enhancement for treating both tumors and their metastasis. The best local radiation therapy enhancement is expected to arise from MNPs irradiated with 10–80 keV photons, due to the much higher mass absorption coefficient of metals relative to biological tissue in this range [1]. In treatments with external beams, this amplification of local dose remains restricted to superficial tumors, because of the limit of ~50 mm in the penetration depth of such X-rays in biological tissue [7]. It has been suggested that this restriction could be circumvented, if the MNP were to be delivered bound to a radioactive isotope into deep cancer sites via targeted radionuclide therapy [155,156] or convention enhanced delivery [104,157]. In some cases, such carriers already use metal ligands or MNPs to bind the radioisotope to the targeting compound [158,159]. In patient treatments with targeted high-Z electron emitters, the radionuclide decay creates a cascade of ejected short-range Auger electrons [159,160,161]. If combined with MNPs or embedded within gold nanocage structures, these Auger electrons are expected to produce a further high-density distribution of LEEs [156,162].

Local amplification of radiation damage poses considerable challenges in the modeling of nanoscopic dose enhancement induced by particle or high-energy photon interaction with MNPs. Following the primary interaction of incident high energy radiation, the description of the electron and photon flux produced within and outside the MNP must include several factors related to initial energy spectrum of all generated Auger and SEs. As explained by Emfietzoglou and Incerti [163], this requires consideration of the electronic structure of the MNP atoms (excitation levels, ionization shells), their de-excitation process (e.g., the Auger electron cascades) as well as a fairly accurate description the electron distribution emitted outside the MNP. An adequate description of the emitted particle flux requires event-by-event simulation of the transport of SEs within the MNP, considering the various energy-loss processes related to their generation. Then, to estimate the nanoscopic dose enhancement, such calculations must be followed by simulations of the transport of electrons emitted in the surrounding biological medium. This is a multifaceted problem, which requires considerable knowledge of all ionization, excitation, and TA formation processes in a complex medium. Although considerable efforts have been made to generate condense-phase cross sections for LEE-induced damage and scattering from biomolecules and DNA [164], this last step remains predominantly limited by an adequate description of the subpicosecond events in biological media. For example, the formation of Tas poses a considerable challenge in Monte Carlo codes, due to the difficulty in representing the quantum physics involved, particularly the time delay caused by retention of scattering LEEs at various sites and electron diffraction [117,165,166].

Another aspect of cancer treatment with MNPs is related to their ability to emit UV photoelectrons in the low energy (0–5 eV) range. Their therapeutic value seems to have been underestimated, possibly because the energy of such electrons is lower than that required to break chemical bonds within DNA. As explained in this article, 0–5 eV electrons photoemitted in the vicinity of MNPs that can induce SSBs in nearby DNA, and even DSBs, when a Pt-CA is bound to the molecule. The reported experiments clearly show that such electrons interact strongly with DNA via the formation of TAs and hence could produce lethal DNA damage via multiple hits, or with single collisions in the presence of CAs. Research combining UV radiation with CAs bound to DNA may thus lead to developments in chemo-phototherapy, capable of eliminating unwanted cells accessible near the body surface, or within certain organs via fiber optics [167].

More generally, multidisciplinary investigations on the production of LEEs by UV, X-rays and fast charged particles interacting with MNPs in biological media should help determine the ensuing nanoscopic enhancement of chemical and biological damage, and thus contribute to the development of more efficient cancer treatments. Future exploitation of the damage induced by LEEs generated around irradiated metals, as well as the general mechanisms of their interaction with DNA should help optimize the design and development of MNPs for antineoplastic applications.

## Figures and Tables

**Table 1:** Work functions (WFs) for different substrates and nanoparticles in vacuum, specific gaseous environments, and water. The WF for Ta covered with bacterial DNA is also included. In the last two columns, the energy range of photoelectrons emitted by 100–280 nm UV radiation and the maximum wavelengths to produce a 0 eV electron in the corresponding environment are given for each substrate or nanoparticle, respectively.

Nanoparticle or Substrate Material ^a^	References	WF in Vacuum (eV)	WF (eV) in Water ^b^	Energy Range (eV) of Photoelectrons for 100–280 nm UV ^c^	Maximum Wavelength (nm) for LEE Emission ^c^
Silver	[39]	4.73	2.15	2.28–10.2	577
Gold	[40]	5.35–5.76	2.26 ± 0.10	2.17–10.1	548
Platinum	[40]	5.12–5.93	2.12 ± 0.09	2.31–10.2	584
Copper	[39]	4.53–5.1	2.08 ± 0.08	2.35–10.3	596
Silicon	[39]	4.85	2.01	2.42–10.4	617
Tantalum	[41]	4.1	3.7 (DNA)	0.73–8.7	335
Silver NP (30 nm)	[42]		4.55 (N_2_–O_2_ mixture)	0–7.8	272
Gold NP (10 nm)	[43]		3.6 (air)	0.83–8.8	344
PbS (9.8 nm)	[44]		4.72 (Ar)	0–7.6	262
CdSe (6 nm)	[45]	4.1–4.5			
FeS_2_	[46]		4.64–4.70 (N_2_)	0–7.7	267
MoO_x_ (x < 3)	[47]		6.6 (O_3_)	0–5.8	187

^a^: For nanoparticles (NPs) the diameters are given in parenthesis. ^b^: Environments other than water are indicated in parentheses. These values consider the complex structure of water in the vicinity of the metal surface. ^c^: The energies and wavelengths are given for the NP or substrate in the corresponding environment.
